# Atrial Fibrillation With Decompensated Heart Failure Complicated With Non-ST Elevation Myocardial Infarction

**DOI:** 10.7759/cureus.21050

**Published:** 2022-01-09

**Authors:** Eman E Shaban, Ahmed E Shaban, Atef Shokry, Haris Iftikhar, Hany A Zaki

**Affiliations:** 1 Cardiology, Aljufairi Diagnostic and Therapeutic Hospital, Doha, QAT; 2 Internal Medicine, Mansoura General Hospital, Mansoura, EGY; 3 Internal Medicine, Mansoura University Faculty of Medicine, Mansoura, EGY; 4 Emergency Medicine, King Abdulaziz University Hospital, Jeddah, SAU; 5 Emergency Medicine, Hamad Medical Corporation, Doha, QAT

**Keywords:** myocardial infarction, prognosis, new-onset, decongestive heart failure, high troponin-t, non-st elevation myocardial infraction, atrial fibrillation

## Abstract

Non-ST-elevation myocardial infarction (NSTEMI) has a less severe ratio of acute coronary syndromes compared with ST-segment elevation myocardial infarction (STEMI), arising from complete occlusion of a major coronary artery. The name implies a syndrome that does not exhibit the dramatic ST-elevation seen in the traditional 12-lead ECG in chest pain patients with a confirmed diagnosis of STEMI. The crucial clinical significance of NSTEMI is that delay in diagnosis can lead to increased morbidity, risk of arrhythmia, and death. It was recently reported that atrial fibrillation (AF) correlates with the risk rise of myocardial infarction (MI), although the mechanism underlying this association is currently unknown. Does atrial fibrillation with decompensated heart failure (DHF) get complicated with NSTEMI? In this article, we describe the case of a 77-year-old male patient diagnosed and admitted as NSTEMI complicated by DHF.

## Introduction

Atrial fibrillation (AF) is more of an electrical complication. It is observed mainly in patients with acute myocardial infarction (AMI). According to the literature, the incidence ranges from 6% to 19% [1]. But then, most of these patients were found to have pre-existing AF, with the incidence of the new-onset atrial fibrillation being as high as 5% [2-3].

Various studies have shown that there is a link between atrial fibrillation and increased mortality (immediate, long-term and short-term) as well as adverse events like a cerebrovascular accident (CVA) and heart failure (HF) [1,4-6]. It is important to note that the pathogenesis of new-onset atrial fibrillation in AMI patients is acute left atrial dilatation caused by high atrial pressure associated with an underlying factor [7]. It may be seen secondary to complications occurring after AMI, but its prognosis is poor when it occurs independently [8].

Non-ST-elevation myocardial infarction (NSTEMI), as the name implies, is myocardial infarction. NSTEMI is frequently associated with ST-segment depression and T wave changes but can occur with normal ECG. Typically, chest pain patients without significantly abnormal ST elevation are closely monitored until other examinations confirm an acute infarction. A typical confirmation is elevated troponin levels. It is also worth mentioning that troponin elevation indicates injury to the myocardial cells, which may be triggered by inflammation trauma, tachycardia, ischemia, strenuous exercise, release or infusion of catecholamines, renal failure, or imbalance in the autonomic nervous system [9].

Atrial fibrillation is considered a significant public health concern owing to its rising prevalence and strong association with poor prognosis. Currently, there are at least 2.7 to 6.1 million individuals with AF in the United States, and there are indications that this figure may double by 2050 [10-12]. AF, a known risk factor for stroke [13,14], has also been a risk factor for myocardial infarction [15].

Atrial fibrillation with decompensated heart failure (DHF) is believed to be complicated by NSTEMI. We describe the case of a 77-year-old male patient diagnosed and admitted as NSTEMI complicated by DHF.

## Case presentation

A 77-year-old male patient was presented to the emergency department with shortness of breath since early morning. The patient had reported a reduction in his urine output recently. He denied any chest pain, palpitation or dizziness, no fever, or significant respiratory symptoms, or lower limb swelling. The patient had been admitted to the hospital because of acute onset shortness of breath associated with orthopnea and paroxysmal nocturnal dyspnea. The patient was known to have diabetes mellitus type II, hypertension, paroxysmal atrial fibrillation on warfarin, osteoarthritis of the knee, multiple myeloma (IgA Kappa) stage III B, recurrent anemia, requiring blood transfusion, previous admissions with volume overload, and end-stage renal disease on peritoneal dialysis.

We took the patient’s vitals and obtained the following results: Temperature 36.3°C (oral), respiration rate (RR) 20 breaths per minute, blood pressure 115/89, oxygen saturation (SpO2) 98%, weight 70 kg.

**Figure 1:**
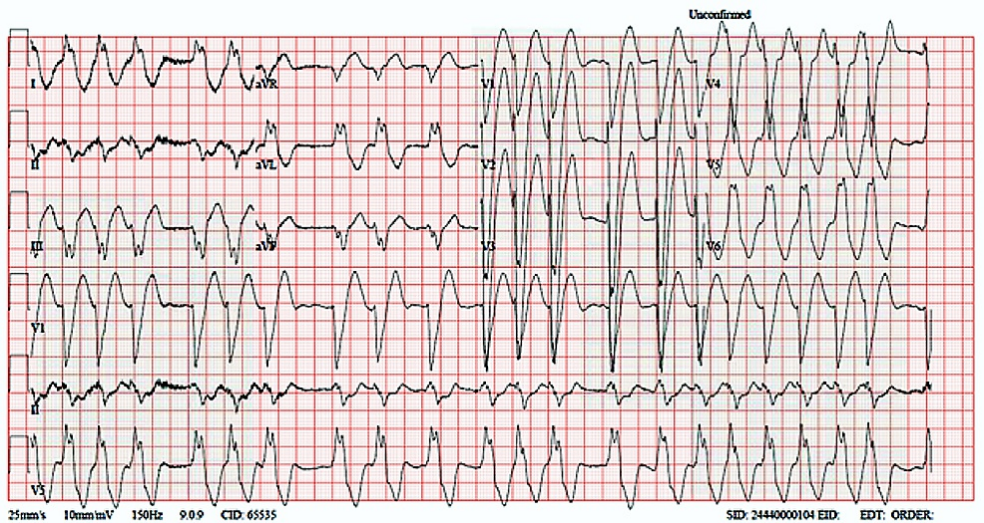
Electrocardiogram (ECG) showed atrial fibrillation with wide complex with old left bundle branch block (LBBB).

**Figure 2:**
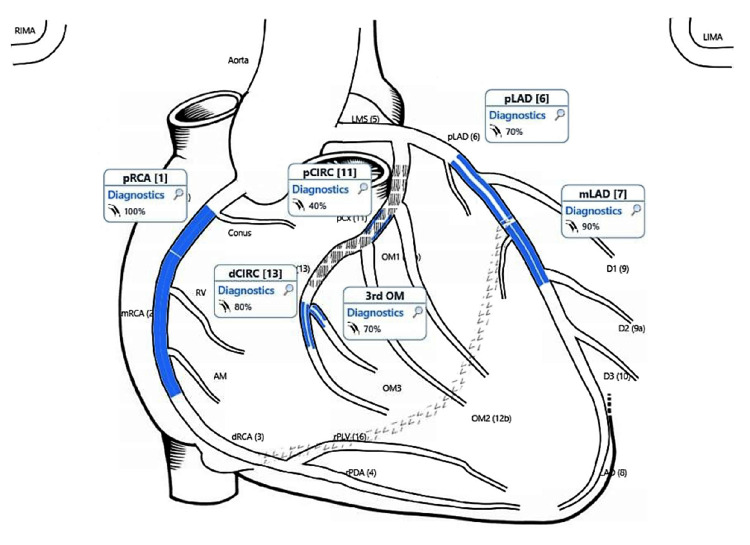
Coronary Angiography. Dominance: Right Dominant, LMS: Normal, LAD Proximal LAD: 70% stenosis followed by Mid LAD: 90% stenosis. Proximal LCx: 40%. There is in-stent restenosis (DES). Distal LCx: 80% stenosis. OM3: 70% stenosis. Proximal RCA: 100% occluded. The distal RCA was supplied by collateral flow from left system (good collateral filling). LMS: Left Main Stem, DES: Drug Eluting Stent, LAD: Left Anterior Descending, LCx: Left Circumflex, RCA: Right Coronary Artery, OM3: Obtuse Marginal Branch-Third

General examination revealed that the patient was in a state of consciousness, oriented, looking well, and not in distress. Cardiovascular examinations first and second heart sounds were normal and showed no added sound. Chest examination revealed bilateral basal fine scattered crepitus. Examination of the abdomen showed it was soft, lax, no tenderness. Lower limb examination revealed mild pitting edema of both limbs from the toes up to the whole of both legs, without any signs of deep venous thrombosis. Figure 1 shows a representation of atrial fibrillation with a wide complex with left bundle branch block (old). The patient was found to have acutely progressive raised troponin (Table 1). As such, he was admitted as NSTEMI complicated by DHF. Clinicians performed a Holter monitor from outpatient clinic recently and found that the patient had been in continuous atrial fibrillation since a few days before Emergency admission, with episodes of fast ventricular response. We, therefore, started on full anti-ischemic management (loaded with heparin infusion, statins, and dual antiplatelet therapy). The patient was seen by the nephrologist, and some change was done over his peritoneal dialysis regime. Three days later, the patient was moved to the heart hospital to continue treatment and seek further therapeutic options. The patient also received 1 unit of packed RBC because of the recurrent anemia, seen by the hematologist, and cleared for coronary angiogram (CAG) while multiple myeloma medication was suspended. CAG was done and revealed three diseased vessels (Figure 2).

**Table 1:** Troponin-T results during the 10-day hospital stay.

Date	Troponin-T
15^th^ October	1979 ng/L
16^th^ October	2906 ng/L
17^th^ October	3902 ng/L
18^th^ October	4264 ng/L
19^th^ October	3827 ng/L
20^th^ October	2962 ng/L
21^st^ October	1895 ng/L
22^nd^ October	1389 ng/L
23^rd^ October	866 ng/L
24^th^ October	594 ng/L

**Figure 3:**
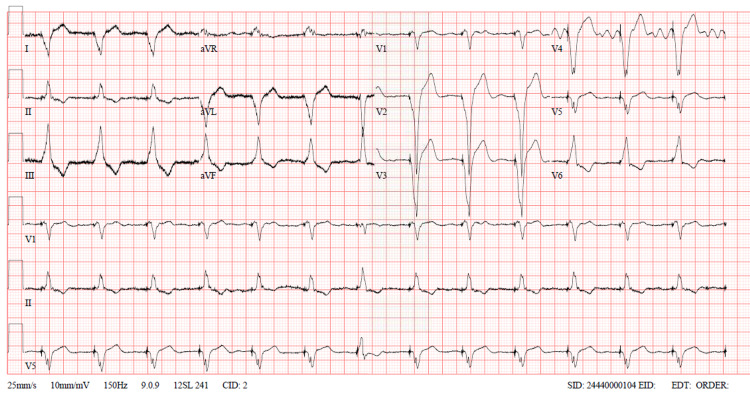
Electrocardiography (ECG) 10 days post-hospital admission showed atrial-sensed ventricular-paced rhythm with prolonged atrio-ventricular conduction, and rate control with ventricular rate 80 beats per minute.

Aspirin treatment was stopped, and warfarin resumed with bridging heparin. The international normalized ratio (INR) has been followed daily until the day of discharge. Upon discharge, the patient’s condition had significantly improved with a drastic reduction of the troponin T (Table 1), with heart rate controlled confirmed by the following ECG (Figure 3).

## Discussion

Myocardial infarction is one of the most common health conditions in developed, developing, and underdeveloped nations. The occurrence of atrial fibrillation in an acute myocardial infarction setting is, therefore, a common phenomenon. Many studies have reported high immediate, long-term, or short-term mortality and even thromboembolic conditions such as ischemic heart disease, ischemic stroke, and atrial fibrillation, reduced quality of life, and reduced capacity to exercise in patients with acute myocardial infarction complicated by atrial fibrillation [1,4-6,16]. The patient, in this case, was admitted not only as an NSTEMI but complicated by DHF, has been found with acutely progressive raised, elevated troponin.

There are a couple of explanations that account for the absence of abnormal ST elevation in acute myocardial infarction. Among these are the possibilities that the patient may be having relatively small infarcts in areas that are not strongly sensed by the lead fields of the 12-lead ECG or probably due to the slow development of the infarct. This also does not imply that the QRS waves and ST-T waves of the ECG do not undergo dynamic changes during the infarct’s time course. At the cellular level, acute ischemia triggers changes in amplitude of action potential, as well as the duration, sloping, or triangularization of the plateau, and in many cases, reduced action potential upstroke velocity in the cells that have been affected. As such, the ECG reveals a dynamic progression of changes in slope and ST level as well as morphology and amplitude of T wave, alongside a widened QRS as a consequence of conduction slowing - all with varying time course and magnitude. All of these may present in NSTEMI, although in a more subtle form than in ST-segment myocardial infarction, in which there are definite elevations of ST segments. As such, any method that is sensitive to changes in QRS and ST-waveform or quantitates them should improve the sensitivity of the infarction test and also reduce the time required for the diagnosis of the syndrome.

The results and analysis from this study, according to findings from recent studies that atrial fibrillation is a risk factor for myocardial infarction [15]. But more important is the fact that we now understand from this case the effect of myocardial infarction type and techniques of AF diagnosis on the MI and AF association. This case illustrates that the link between MI and AF is limited to NSTEMI. Of course, this may shed some light on the underlying mechanism that serves as the link between AF and MI. It is important to note that ST-elevation myocardial infarction (STEMI) and NSTEMI share similar long-term prognosis. However, they have significantly different pathophysiology and treatment [17,18]. The difference in treatment strategies stems from the fact that the culprit artery in STEMI is occluded by a thrombus in most cases, while in NSTEMI, the culprit artery is patent with a non-occlusive thrombus. Considering this and given the fact that there is an association between AF with NSTEMI, it may mean that direct coronary thromboembolization may not necessarily be the primary mechanism by which atrial fibrillation leads to myocardial infarction. This accords with the general belief that direct thromboembolization is not so common due to the anatomical barriers that impede the chances of direct coronary embolization, such as the differences between the caliber of the coronary arteries and aorta, location of the coronary vessels at the aortic root, the emergence of coronary arteries at the right angle, and the fact that most of the coronary filling occurs during diastole [19].

It is also worth mentioning that there may be more mechanisms that contribute to the development of congestive heart failure in patients experiencing non-ST elevation acute coronary syndromes (ACS). Diabetes and advancing age are established, independent, and very powerful risk factors for atherosclerosis development, and there is the possibility that the association observed between diabetes and age and the risk of chronic heart failure (CHF) development may be due to a serious underlying disease of the coronary artery in these patients. But then, both diabetes and advancing age are also associated with diastolic dysfunction, and this may play a role in association with the development of CHF [20].

## Conclusions

Atrial fibrillation is associated with a high risk of incident myocardial infarction. This association differs based on the MI type. Atrial fibrillation increases the risk of NSTEMI but does not influence STEMI. Once two or more critically cardiac diseases have been diagnosed simultaneously, they must be treated side-by-side on time, as highlighted in the case report with anticoagulation, close monitoring, and AF treatment considering AMI at intervals.
